# Comment on “Unveiling Resmetirom: A Systematic Review and Meta‐Analysis on Its Impact on Liver Function and Safety in Non‐Alcoholic Steatohepatitis Treatment”

**DOI:** 10.1002/jgh3.70446

**Published:** 2026-07-20

**Authors:** Rajeh Ruzayqat

**Affiliations:** ^1^ Faculty of Medicine Hebron University Hebron Palestine

To the Editor,

1

We read with interest the article by Hashim et al., which evaluated the effects of resmetirom in non‐alcoholic steatohepatitis (NASH) through a systematic review and meta‐analysis. The authors included three randomized controlled trials in the quantitative synthesis and presented multiple forest plots to summarize the findings [1].

According to Figure 1, the PRISMA flow diagram reports “Records after duplicates removed (*n* = 353).” However, the total number of identified records (321 from databases and 32 from other sources) already equals 353. This suggests that the reported number likely reflects records prior to deduplication rather than after. Accurate reporting of the study selection process is essential, and clarification of this discrepancy would improve transparency.

According to the data presented in the Table 1 (columns 4 and 5), the total number of participants sums to 1942 “Resmetirom: 322 (80 mg) 323 (100 mg), 327 (80 mg) 495 (100 mg), 84. Placebo: 32, 318, 41.” However, the Results section reports a total of 2234 participants “Total number of patients among the studies were 2234 including all the sub‐groups.” This discrepancy does not appear to be clearly explained in the study, and no justification is provided for the difference between the reported totals. This raises concerns regarding the accuracy and consistency of sample size reporting.

In addition, the Results section reports a non‐significant effect of resmetirom on AST levels “the AST levels (MD –3.60; 95% CI –7.44 to 0.24; *p* = 0.07) and GGT levels (MD –17.02; 95% CI –35.12 to 1.09; *p* = 0.07) also gave nonsignificant results”. Despite this, the Discussion describes a significant effect on AST “Our results show that Resmetirom play a significant role in LDL‐C and AST levels compared with placebo.” This inconsistency between sections may affect the interpretation of the findings and should be explicitly addressed.

In this context, clarification regarding data reporting, sample size derivation, and outcome interpretation would improve the internal consistency and transparency of the study, ultimately strengthening the reliability of its conclusions.

## Conflicts of Interest

The author declares no conflicts of interest.

## Data Availability

Data sharing not applicable to this article as no datasets were generated or analysed during the current study.
